# Estimation of the Internal Dose Imparted by ^18^F-Fluorodeoxyglucose to Tissues by Using Fricke Dosimetry in a Phantom and Positron Emission Tomography

**DOI:** 10.3389/fnume.2022.815141

**Published:** 2022-02-14

**Authors:** Thititip Tippayamontri, Esteban Betancourt-Santander, Brigitte Guérin, Roger Lecomte, Benoit Paquette, Léon Sanche

**Affiliations:** ^1^Department of Nuclear Medicine and Radiobiology, University of Sherbrooke, Sherbrooke, QC, Canada; ^2^Centre Hospitalier Universitaire de Sherbrooke (CHUS) Research Center, Faculty of Medicine and Health Sciences, University of Sherbrooke, Sherbrooke, QC, Canada; ^3^Department of Radiological Technology and Medical Physics, Faculty of Allied Health Sciences, Chulalongkorn University, Bangkok, Thailand; ^4^Departement of Radiotherapy, National Institute of Cancer, Santiago, Chile; ^5^Sherbrooke Molecular Imaging Center, Centre de recherche du CHUS (CRCHUS), Sherbrooke, QC, Canada

**Keywords:** internal radiation dose, Fricke dosimeter, 18F-FDG, positron emission tomography, phantom

## Abstract

**Purpose:**

Assessment of the radiation dose delivered to a tumor and different organs is a major issue when using radiolabelled compounds for diagnostic imaging or endoradiotherapy. The present article reports on a study to correlate the mean ^18^F-fluorodeoxyglucose (^18^F-FDG) activity in different tissues measured in a mouse model by positron emission tomography (PET) imaging, with the dose assessed *in vitro* by Fricke dosimetry.

**Methods:**

The dose-response relationship of the Fricke dosimeter and PET data was determined at different times after adding ^18^F-FDG (0–80 MBq) to a Fricke solution (1 mM ferrous ammonium sulfate in 0.4 M sulfuric acid). The total dose was assessed at 24 h (~13 half-lives of ^18^F-FDG). The number of coincident events produced in 3 mL of Fricke solution or 3 mL of deionized water that contained 60 MBq of ^18^F-FDG was measured using the Triumph/LabPET8^TM^ preclinical PET/CT scanner. The total activity concentration measured by PET was correlated with the calculated dose from the Fricke dosimeter, at any exposure activity of ^18^F-FDG.

**Results:**

The radiation dose measured with the Fricke dosimeter increased rapidly during the first 4 h after adding ^18^F-FDG and then gradually reached a plateau. Presence of non-radioactive-FDG did not alter the Fricke dosimetry. The characteristic responses of the dosimeter and PET imaging clearly exhibit linearity with injected activity of ^18^F-FDG. The dose (Gy) to time-integrated activity (MBq.h) relationship was measured, yielding a conversion factor of 0.064 ± 0.06 Gy/MBq.h in the present mouse model. This correlation provides an efficient alternative method to measure, three-dimensionally, the total and regional dose absorbed from ^18^F-radiotracers.

**Conclusions:**

The Fricke dosimeter can be used to calibrate a PET scanner, thus enabling the determination of dose from the measured radioactivity emitted by ^18^F-FDG in tissues. The method should be applicable to radiotracers with other positron-emitting radionuclides.

## Introduction

In all preclinical and clinical applications of radiotracers, a major parameter, which should be known and controlled to minimize side effects, is the energy imparted per unit mass (i.e., the radiation dose) by the energetic primary photons or fast charged particles to different tissues and organs (1–3). Previous studies reported biological effects such as cancer and genetic defects, and in some cases, the cause of lethality, resulting from radiation exposure following administration of radiotracers (3, 4). Moreover, positron emission tomography (PET) scans deliver one of the highest effective radiation doses to patients (0.019 to 14.1 mSv/MBq) when compared to other nuclear medicine procedures (5, 6). As a result, it becomes a challenging issue of radiation safety for internal radiation exposure from the radiotracers. Although predictable and accurate radiation doses can be estimated when delivered by exposure to external radiation (3, 4), it is much more difficult to calculate or measure doses from internal radiation sources (5, 6). The assessment of the radiation dose from radiotracers delivered to malignant cells and different normal tissues still remains a major issue in diagnostic, and even more so, in radiation molecular targeted procedures. Since local tumor treatment in radionuclide targeted therapy can improve radiation dose deposition in the tumor tissue while minimizing the radiation dose to surrounding normal tissues (7), there is presently a pressing need to develop reliable methods to accurately estimate the dose arising from internal radiation sources. Furthermore, direct intratumoral injection of radiopharmaceuticals has shown its efficacy and initial promise in animal models and a few clinical cases (8, 9). This type of administration approach could outperform the efficacy of systemic targeted radionuclide therapy. While the use of intratumoral delivery of radiopharmaceutical is increasing, it is imperative to be able to verify the accuracy of absorbed dose in the tumor tissue as well as in nearby normal tissues.

The radionuclides used in diagnostic PET imaging include ^18^F, ^15^O, ^13^N, ^11^C, ^64^Cu, ^68^Ga, ^82^Rb, ^89^Zr and ^124^I. ^18^Fluorine (^18^F)-radiotracers (e.g., ^18^F-FDG, ^18^F-FLT, ^18^F-MISO, ^18^F-NaF, and many others) (10, 11) are routinely administered in many research protocols and clinical studies (e.g., cancer diagnosis and treatment follow up) (12, 13). Presently, ^18^F-FDG is the commonly used tracer. ^18^F-FDG PET has been proven to be a sensitive and reliable imaging modality for detection, staging/ restaging, and therapy response assessment in oncology. ^18^F-FDG PET provides essential information for radiation treatment planning, helping in critical decisions, particularly when delineating tumor volumes (14).

PET is a quantitative imaging technique. Positron-emitting radiotracers emit short-range (≤ 1 mm to a few mm in water) positrons that deposit energy along their paths (15). At the end of their tracks, they annihilate, generating two 511 keV photons traveling in opposite directions that can be detected by a PET scanner. The total activity of radiotracers in the different organs and the dose can be estimated by computer simulations, e.g., with the MIRD formalism (16) or OLINDA/EXM software (17). However, these evaluations of the dose may be limited by the Monte Carlo simulations, which are sensitive to voxel size effects, and depend on interaction cross-sections and source design (18). Regardless of the site being imaged, injection of a PET radiotracer results in systemic uptake and radiation exposure (19). The dose imparted to the target tissues, as well as to other organs and tissues, critically depends on the pharmacokinetics of the radiotracers, and on the physical decay scheme of the radionuclide administered.

There are several approaches that have been developed for measuring absorbed doses from ionizing radiation (20). Among these methods, the Fricke dosimeter can be used to determine the dose without reference to another dosimeter. The Fricke dosimeter is the most widely used as a chemical dosimetry primary standard. It is based on the oxidation of ferrous ions to ferric ions, caused by the formation of the free radicals when the solution is irradiated by the ionizing radiation (21–23). The Fricke dosimeter allows accurate measurements of large radiation doses imparted by external beam irradiation from radionuclide sources and particle accelerators. However, little has been published on the estimation of radiation doses from radiotracers using chemical procedures. There is a report on the feasibility of using a ferrous sulfate-benzoic acid-xylenol orange (FBX) dosimeter to measure the dose from radionuclide solutions of ^99m^Tc and ^131^I (24). The FBX dosimeter exhibited a linear dose response as a function of activity. Considering the widespread use of PET in diagnostic medicine and the accuracy of the Fricke dosimeter, there is considerable interest in the evaluation of doses from positron emitting sources with a chemical dosimeter. To test the viability of such methods, we evaluate the dose administered by ^18^F-FDG using the chemical Fricke dosimeter. We measure the yields of ferric ions induced by free radicals in a Fricke solution produced by ^18^F-FDG irradiation. The proposed method can be beneficial to evaluate the radiation absorbed dose after intratumoral administration of ^18^F-FDG, where self-dose is the main contributor to the overall absorbed dose. This work aims to estimate the self-dose to a tissue from a uniform distribution of non-penetrating radiation by measuring the cumulative activity in the tissue and multiplying it by the dose-per-disintegration as measured using quantitative imaging of a Fricke dosimeter.

Since it is the photons from the annihilation of positrons that are detected by a PET scanner, the mean accumulated activity of the radiotracer in different tissues measured by PET could potentially be used as a surrogate of the dose if a relationship between the activity measured by PET and the dose measured by the Fricke dosimeter can be established. In other words, a chemical dosimetry standard can be developed by the calibration of a transfer dosimeter in a total absorption experiment, and subsequently apply the transfer dosimeter in a water phantom, under reference conditions. The purpose of the present study is to evaluate the feasibility of using the Fricke dosimeter for estimating the radiation dose in patients during PET imaging with positron emitters. Here, we can further relate the dose absorbed (Gy) in different tissues to radioactive activity (Bq) and counts from the PET imaging data. ^18^F-FDG was selected for its widespread use in clinical oncology. In our experiments, the response of the Fricke solution was first determined by using the total absorption of external gamma radiation. Next, the dose absorbed by the system with reference to ^18^F-FDG activity was obtained using the Fricke dosimeter as the transfer dosimeter. All measurements of the doses were performed in a three–dimensional environment, i.e., Fricke dosimetry and PET imaging. We used a 3 mL of subject volume, which is enough solution to measure the change in the optical absorbance in the Fricke dosimeter. Combined with PET imaging, this procedure provides individual cumulated activities with good accuracy. Moreover, to investigate the feasibility of Fricke dosimetry in estimating internally absorbed doses, we performed animal PET imaging of the ^18^F-FDG biodistributions and dosimetry calculations with the MIRD schema.

## Materials and Methods

Three groups of study were performed: i) positive controls, which refers to Fricke solution alone plus gamma irradiation, ii) negative controls, which refers to Fricke solution containing non-radioactive FDG plus gamma irradiation, and iii) experimental conditions, which refers to Fricke solutions that contained various concentrations of ^18^F-FDG.

### ^18^F-FDG Production

^18^F-FDG (CYCLODX) was prepared following usual methods at the Sherbrooke Molecular Imaging Center (CIMS), CIUSSS de l'Estrie - Centre Hospitalier Universitaire de Sherbrooke, Canada). Table 1 shows the chemical components of the ^18^F-FDG solution. Typical molar activity of ^18^F-FDG is about 63.3 GBq/nmol with a total quantity of 1.6 nmol (98.8 pmol/mL) of ^18^F-FDG.

**Table 1:** The chemical components in ^18^F-FDG preparation.

**Ingredient**	Quantity Per Batch (Initial activity ≥140 GBq ^**18**^F)^*^
^18^F-FDG at end of synthesis	90–180 GBq
Citrate buffer solution (Ph Eur)	6 mL
Volume of sodium chloride solution 0.9%	16.5 mL of saline added to bulk finished product vial prior to dispensing
Water for injection	10 mL
Total volume	33 ± 1.5 mL

* *Quantities indicated are calculated values*.

### Fricke Dosimeter: Principles of Preparation and Dose Measurement

The Fricke dosimetry relies on measuring the ferric ions (Fe^3+^) produced as a by-product of water radiolysis [e.g., hydrogen radical (H^•^), hydroxyl radical (^•^OH), hydrated electron (e_aq_) and hydrogen peroxide (H_2_O_2_)] through the oxidation of ferrous ions (Fe^2+^) in a solution. The mechanism for the radiolytic oxidation of Fe^2+^ to Fe^3+^ ions in the Fricke dosimeter is well-understood and the rate constants at 25 °C of the individual reactions taking place are well-known (22).


$$\begin{array}{l} \begin{array}{cc} {\text{e}}_{\text{aq}}^{-}+{\text{H}}^{+}\to {\text{H}}^{.} & \left(\text{k}\sim 1.12x10^{10}\;{\text{m}}^{-\text{1}}{\text{s}}^{-\text{1}}\right)\\ {\text{H}}^{.}+{\text{O}}_{2}\to {\text{HO}}_{2^{.}} & \left(\text{k}=2.1x10^{10}\;{\text{m}}^{-\text{1}}{\text{s}}^{-\text{1}}\right)\\ {\text{Fe}}^{2+}+{\text{H}}^{.}\to {\text{Fe}}^{3+}+{\text{H}}_{2}+{\text{OH}}^{-} & \left(\text{k}=1.3x10^{7}\;{\text{m}}^{-\text{1}}{\text{s}}^{-\text{1}}\right)\\ {\text{Fe}}^{2+}+.\text{OH}\to {\text{Fe}}^{3+}+{\text{OH}}^{-} & \left(\text{k}=3.4x10^{8}\;{\text{m}}^{-\text{1}}{\text{s}}^{-\text{1}}\right)\\ {\text{Fe}}^{2+}+{\text{HO}}_{2^{.}}\to {\text{Fe}}^{3+}+{\text{HO}}_{2^{-}} & \left(\text{k}=7.9x10^{5}\;{\text{m}}^{-\text{1}}{\text{s}}^{-\text{1}}\right)\\ {\text{HO}}_{2}-+{\text{H}}^{+}\to {\text{H}}_{2}{\text{O}}_{2} & \left(\text{k}\sim 2.66x10^{10}\;{\text{m}}^{-\text{1}}{\text{s}}^{-\text{1}}\right)\\ {\text{Fe}}^{2+}+{\text{H}}_{2}{\text{O}}_{2}\to {\text{Fe}}^{3+}+.\text{OH}+{\text{OH}}^{-} & \left(\text{k}=52\;{\text{m}}^{-\text{1}}{\text{s}}^{-\text{1}}\right) \end{array} \end{array}$$

The yield of Fe^3+^ ions in an irradiated Fricke dosimeter is expressed in terms of the primary products of the radiolysis of the solution according to the equation of G(Fe^3+^) = 3G(H^**.**^) + 2G(H_2_O_2_) + G(^**.**^OH) (22). The increase in Fe^3+^ concentration results in a change in optical density (OD), which can be measured by a spectrophotometer. The yield of Fe^3+^ can consequently be related to the dose delivered to the Fricke solution.

The Fricke dosimeter solutions were prepared using ammonium ferrous sulfate hexahydrate (NH_4_)_2_Fe(SO_4_)_2_.6H_2_O (99.99%, Sigma Aldrich), sulfuric acid (98%, Sigma Aldrich), as received and deionized water (Baxter, Canada). During the preparation of the solutions, special attention was taken to minimize impurities. Standard radiation chemistry procedures were carefully followed in cleaning glassware having been in contact with the solution to be irradiated (22). The air-saturated Fricke solutions were prepared with minimum exposure to light; the solute consisted of 1 mM Fe^2+^ ions in 0.4 M H_2_SO_4_. No chloride was added (25).

#### Fricke Solution: Gamma-External Beam Irradiation

To validate the accuracy and linearity of the dosimeter, the Fricke solution without and with non-radioactive FDG was irradiated by an external gamma beam. All experiments were carried out using ^60^Co γ-rays generated by a calibrated Gammacell 220 (Atomic Energy of Canada Limited). The dose was determined at the center of the Gammacell 220 chamber. The calibration curve was prepared by irradiating samples up to 80 Gy at 25°C, with a dose rate of 0.78 Gy/min. For negative controls, different concentration (0.01–0.16 mM) of non-radioactive FDG were added to the Fricke solution and irradiated with γ-rays.

#### Fricke Solution: ^18^F-FDG-Internal Irradiation

To evaluate the internal radiation dose of ^18^F-FDG, 3 mL of freshly prepared 1 mM Fricke stock solution, to which ^18^F-FDG was added, was divided into separate portions so as to obtain different activities of radioactive ^18^F-FDG, over the range 15-80 MBq in different vials (*n* = 3–5 vials per activity). The ^18^F-FDG activity was measured using a calibrated dose calibrator for nuclear medicine model Capintec^®^ CRC^TM^-35R (Capintec Inc.). A gamma counter was used to detect the presence of ^18^F in the Fricke solution (26). The vials were then gently shaken to have a homogenously mixed solution. The samples of Fricke solution containing different ^18^F-FDG activities were separated from each other. Radioactive and non-radioactive Fricke solution vials were stored at room temperature, in darkness in a shielded safe.

#### Radiation Dose Calculations With the Fricke Dosimeter

After external or internal irradiation, Fricke solutions were transferred into quartz cuvettes to measure the optical density (OD) using a DU530 UV-VIS spectrophotometer (Beckman) at 304 nm. For Fricke solution containing ^18^F-FDG, the OD value was measured at time 0, 30, 60, 110, 240, 350, 430, and 1,450 min after adding ^18^F-FDG. For ^60^Co gamma irradiation, the OD value was immediately measured after irradiation. The OD values were corrected by subtracting the OD of the control (or non-irradiated Fricke solution) from that of the radiolabeled ^18^F-FDG Fricke dosimeter. The control has the OD value of the non-radiolabeled FDG Fricke dosimeter that contained the same amount of FDG (1.6 nmol) as in the radiolabeled ^18^F-FDG Fricke dosimeter. The dose in the Fricke solution was calculated from Equation [1] (22). All data were reported as means ± S.D. from triplicate experiments.


(1) $$\begin{array}{l} D=\frac{\Delta OAR_{F}K_{vial}K_{dd}K_{E}}{\varepsilon G\left(Fe^{3+}\right)\rho l} \end{array}$$

where

*D* is the radiation dose in water (Gy);

Δ*OA* is the difference between the optical density of the non-irradiated solution and the optical density of an irradiated solution;

*R*_*F*_ is the ratio of the dose in water to the dose in the Fricke dosimeter contained by the same imaginary walls at the reference point in the phantom (for R_F_ =1.0032);

*K*_*vial*_ is the correction factor that considers the perturbation due to the walls of the Fricke vials (for *K*_*vial*_=1.00);

*K*_*dd*_ is the correction factor that accounts for the non-uniformity of the irradiation field across the diameter of the vial (*K*_*dd*_=1.00);

*K*_*E*_ is the correction factor taking into account any integrity dependence of G(Fe^3+^) (*K*_*E*_=1.00);

ε*G(Fe*^3+^*)* is the product of the molar extinction coefficient and the chemical yield of the Fe^3+^ determined by comparison to calorimetry (ε*G(Fe*^3+^*)* = 3.5060 and 3.498 cm^2^ J^−1^ for ^60^Co γ-rays and ^18^F-FDG irradiation, respectively);

ρ is the density of the Fricke solution at 25°C (ρ= 1.0227 × 10^−3^ kg cm^−3^);

*l* is the optical path length of the spectrophotometer cuvette (*l* = 1 cm).

Note that since the Fricke solution consists of 96% water by weight, the primary products of radiation were mostly those of water radiolysis. Although considered approximative, this process was extensively discussed by Klassen et al., where the *G*-values for a Fricke solution were assumed to behave similarly to those for water (27). The *G(Fe*^3+^*)* value is expected to be approximately energy independent for low-LET radiation (27). Therefore, only *G(Fe*^3+^*)* values for ^137^Cs/ ^60^Co and ^18^F irradiation are different, as indicated in the text above.

### PET Measurements: Principle and Dose Correlation to Fricke Dosimeter

PET radiation detection was performed to indirectly evaluate the dose from positrons emitted from ^18^F-FDG in the 3D and systemic environment of the Fricke solution and animal PET imaging, respectively. Thereafter, the total activity concentration measured by PET was correlated with the calculated dose from the Fricke dosimeter, at any exposure activity of ^18^F-FDG. The linear regression graphs of the relationship of radiation dose measured by the Fricke dosimeter and time-integrated activity detected by PET imaging as a function of administered activity of ^18^F-FDG were performed with fitting parameters of set intercept at the origin (0.0) and forecast (forward and backward) at 0.0 and 0.0, respectively.

#### PET Detection of ^18^F-FDG in the Fricke Solution

**Figure 1:**
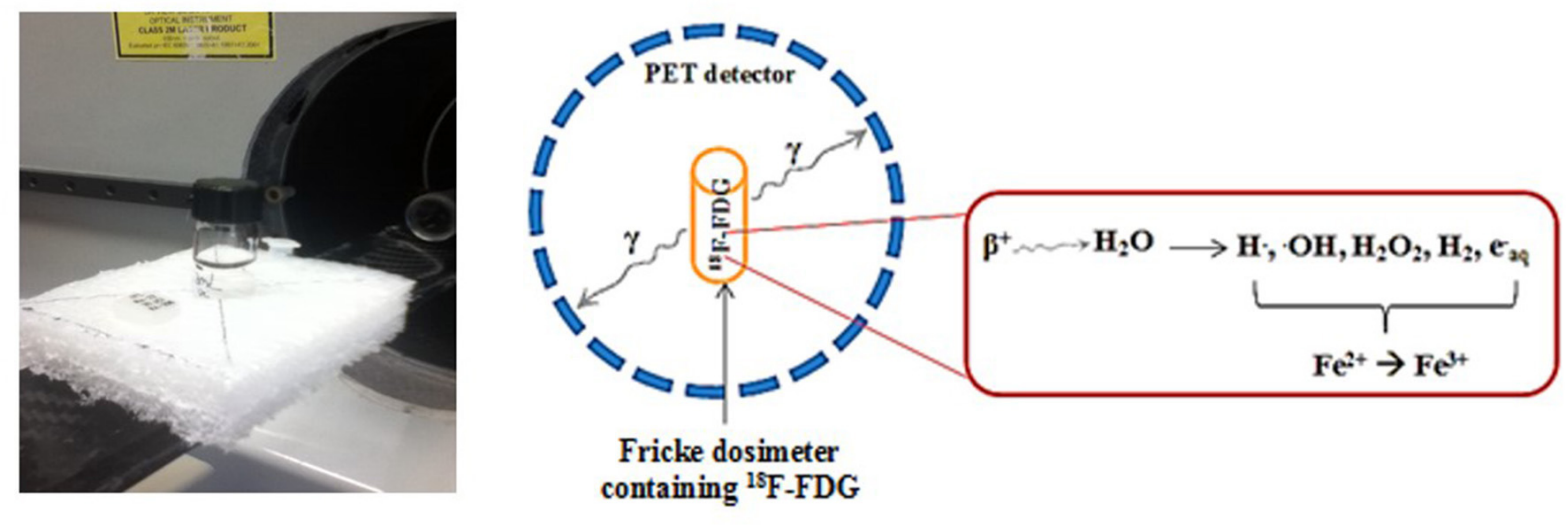
The experimental setup of the ^18^F-FDG radiolabelled Fricke dosimeter with PET scanner. The vial is moved within the scanner field of view for PET imaging.

Figure 1 shows a schematic diagram of the ^18^F-FDG radiolabelled Fricke dosimeter and PET imaging setup. The number of coincident events produced in 3 mL of Fricke solution or 3 mL of deionized water that contained 60 MBq of ^18^F-FDG was measured using the Triumph/LabPET8^TM^ preclinical PET/CT scanner (Gamma Medica, Northridge, CA) available at the CIMS. The characteristics of the LabPET8^TM^ were described by Bergeron et al. (28). PET images of the vials were collected at 0, 1, 2, 3, and 4 h after adding ^18^F-FDG into the Fricke solution. To compensate for the lower ^18^F-FDG activity, PET scanning times were 3.00, 5.12, 9.50, 16.24, and 30.06 min, respectively. A single CT imaging of the vials was performed at the end of the PET scan for attenuation correction of the emission data. The OD and radioactivity were measured prior and after the entire scanning procedure mentioned above. The raw data were reconstructed using a maximum-likelihood expectation-maximization (ML-EM) algorithm implementing the physical modeling of the detector response functions. All PET images were corrected for the physical radionuclide decay, dead time and differences in crystal detection efficiency. Region of interest (ROIs) analysis was carried out with the built-in function in the LabPET image analysis software. The radioactivity in the subject vial (Fisher brand 15 × 45 mm, 1DR, Fisher Scientific, ON) was obtained as cps/mL from reconstructed PET images. To obtain quantitative radioactivity data, the PET system was calibrated by acquiring data from an in-house fabricated phantom that mimics a mouse model filled with an ^18^F-FDG solution of known radioactivity. A cylindrical phantom (25.7 mL) containing 2.02 MBq of ^18^F-FDG was used to obtain a calibration factor for converting the radioactive counts per second into percent injected activity/gram (%IA/g). Thus, the pixel counts of the PET image in cps/mL could be converted into the activity concentration (MBq/mL) by multiplying the ROIs with known added activity of ^18^F-FDG. The relation of total activity concentration measured by PET was used to correlate, with the calculated dose from the Fricke dosimeter, any exposure activity due to ^18^F-FDG.

#### Animal PET Imaging of ^18^F-FDG

Human colorectal HCT116 tumor cells (2 × 10^6^, 0.1 mL) were inoculated subcutaneously (*s.c*.) into each rear flank of outbred male nude mice at 4–6 weeks of age (Charles River Laboratories, Saint-Constant, QC, Canada) (29). Housing and all procedures involving animals were performed according to the protocol approved by the Animal Care and Use Committee. Animals were anesthetised by inhalation of 1.0–1.5% isoflurane and 1.0–1.5 L/min oxygen for radiotracer administration and PET procedures. To evaluate the dose distribution of ^18^F-FDG, animals were respectively injected in the tail vein with a single intravenous (*i.v*.) dose of 10 MBq/100 μL of ^18^F-FDG (*n* = 1), and a single *i.t*. injection of 15 MBq/30 μL of ^18^F-FDG solution into the tumor on one side of the rear flank (*n* = 3), whereas the contralateral tumor was not treated. The administration of ^18^F-FDG by either *i.t*. or *i.v*. injection was performed with the animal placed inside the scanner. Dynamic PET images were recorded from 0 to 120 min post-injection using the LabPET8^TM^ scanner. The raw data were reconstructed using the following parameters: 55-mm image diameter with 120 × 120 × 128 arrays. Images were acquired dynamically over the axial field of view of 75 mm.

### Animal Dosimetry and MIRD Dose Calculation

Radiation doses were estimated from the animal biodistribution data of ^18^F-FDG. To quantify ^18^F-FDG uptake in the tumor and organs, activities were measured by ROI analysis of the whole-body PET images. To obtain quantitative radioactivity data with mice, the PET system was calibrated as described above. The area under the curve, so called time-integrated activity (MBq.h), was determined by trapezoidal integration up to 120 min post injection, assuming that the radiotracer underwent only physical decay with no biological elimination from the source organ.

The total accumulated dose in the tumor tissue and normal organs can be calculated by the following equation:


(2) $$\begin{array}{l} D=A\;\times \;M\;\times \;C \end{array}$$

Where *D* is the dose (Gy);

*A* is the time-integrated activity per gram of tissue (MBq.h/g);

*M* is the tissue mass (g) (Appendix 2);

*C* is the conversion factor of 0.090 Gy/MBq.h, derived from the relationship between Fricke dosimetry and PET imaging (Appendix 1).

This calculated dose of ^18^F-FDG was compared to those calculated using the MIRD program of Bolch et al. (30). The MIRD formulation was applied to calculate the dose of the animal tissues/organs according to:


(3) $$\begin{array}{l} D=\underset{i}{\sum }A_{i}\;\times \;S\text{-Value} \end{array}$$

where

*D* is the target organ dose (mGy)

*A*_i_ is the time-integrated activity in source organ *i* (MBq.h)

*S*-Value is the dose factor mean dose per time-integrated activity in target region (Gy/MBq.h), (Appendix 2). The summation of doses is from each source organ to the target organ.

### Statistical Analysis

Statistical analyses were performed using GraphPad Prism 6.0. The results are expressed as the mean ± standard deviation (S.D.), calculated from triplicate experiments. A *P*-value <0.05 was considered to be statistically significant.

## Results

**Figure 2:**
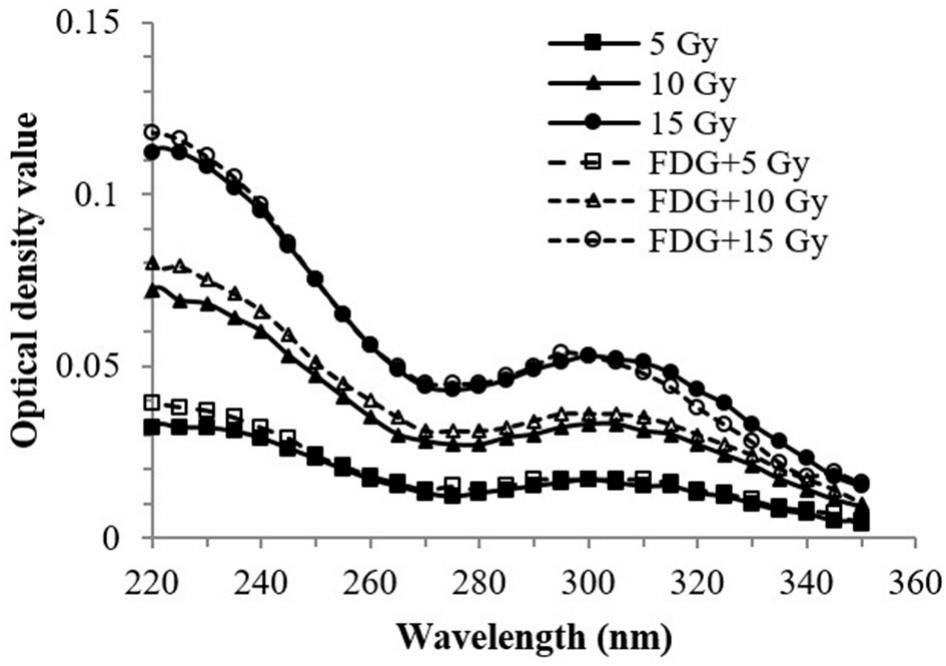
Absorption spectra of the irradiated Fricke solution containing or not non-radioactive FDG. The Fricke solution containing 0.7 ng of non-radioactive FDG (98.8 pmol/mL) was irradiated with 5, 10 and 15 Gy from a ^60^Co gamma source. The optical density (OD) values at 220–350 nm was measured immediately after irradiation.

Absorption spectra of the gamma irradiated Fricke solution, with and without non-radioactive FDG, are shown in Figure 2. Absorption spectra of the irradiated Fricke solution containing non-radioactive FDG were essentially the same as those of the Fricke solution alone. The maximum OD values were observed around 224 and 304 nm, indicating that the maximum absorbance peaks were due to the presence of ferric ions (Fe^3+^) in the solution.

**Figure 3:**
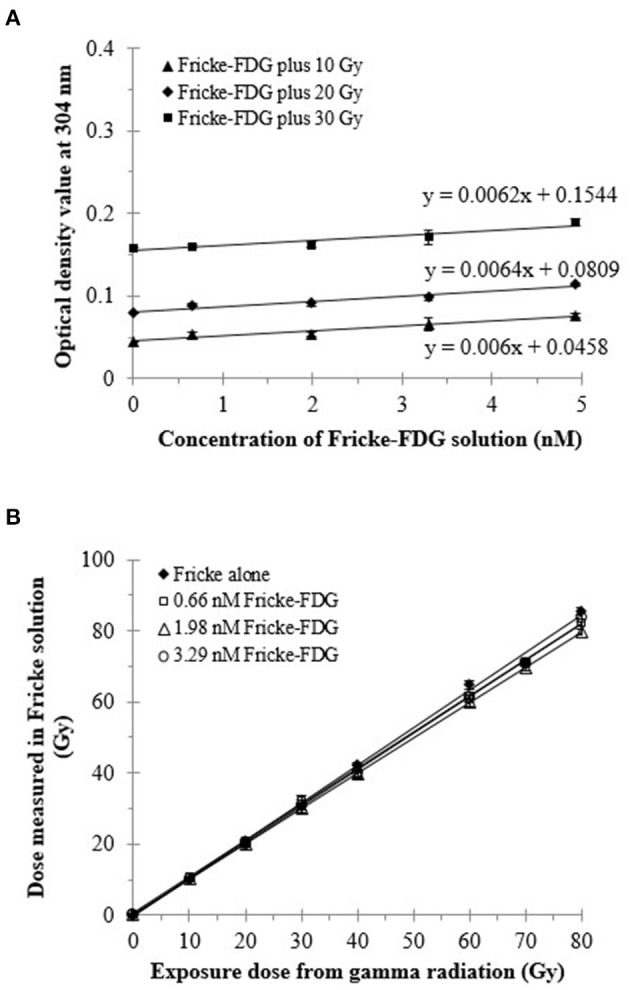
Response of ^60^Co-gamma-ray irradiated Fricke dosimeter containing non-radioactive FDG. **(A)** Changes in the OD of the Fricke solution with different amounts (or concentrations) of non-radioactive FDG. **(B)** Absorbed dose in the irradiated Fricke dosimeter containing 0.4, 1.1, and 1.8 ng of non-radioactive FDG (98.8 pmol/mL) after correction for the presence of FDG on the OD. The OD at 304 nm was measured immediately after irradiation.

Calculated decay activity and measured absorbed dose in the Fricke dosimeter, after addition of various initial activities of ^18^F-FDG are shown in the Table 2. Figure 3A shows the response of the Fricke solution containing non-radioactive FDG after irradiation with 10–30 Gy of gamma ray radiation. The OD values at 304 nm slightly increased with increasing the amount of non-radioactive FDG into the Fricke solution. This suggests that the presence of non-radioactive FDG slightly affects chemical processes in the Fricke dosimeter. Thus, the presence of ^18^F-FDG may affect the reproducibility of the Fricke dosimeter, indicating a possible limitation of this method. Since impurities can cause changes in the OD value of the Fricke solution resulting in lower precision, it is worth considering this possible experimental artifact in the present investigation. Therefore, for calculating a final dose from ^18^F-FDG, the dose obtained from the non-radioactive FDG Fricke solution was subtracted from the dose obtained from the ^18^F-FDG Fricke solution. As shown in Figure 3B, with this correction, a gamma irradiated Fricke solution containing 0.66, 1.98, and 3.29 nM of non-radioactive FDG displayed a similar value of final dose compared to that measured by the standard Fricke dosimeter (or the positive control group). A linear response of the Fricke dosimeter with exposure dose from gamma radiation was observed. After correction for slight modifications from the presence of FDG in the Fricke solution, OD changes can be attributed only to the radiation emitted by the ^18^F-radiotracer.

**Table 2:** Calculated decay activity, measured absorbed dose and percentage of cumulative dose in the Fricke dosimeter, after addition of various initial activities of ^18^F-FDG.

**Time (min)**	**0**	**2**	**30**	**60**	**110**	**240**	**350**	**430**	**1450**
**Initial activity (MBq)**	**Decay activity (MBq)**								
15	15.00	14.83	12.00	10.17	7.32	3.73	1.63	0.96	0.00
20	20.00	19.33	15.46	12.53	9.83	4.48	2.01	1.18	0.00
30	30.00	30.00	25.00	20.22	14.62	7.53	3.34	1.89	0.00
40	40.00	39.53	30.03	26.13	19.23	8.82	3.96	2.31	0.00
60	60.00	59.90	48.74	39.74	31.06	13.67	6.49	3.93	0.00
80	80.00	79.79	64.27	50.96	39.58	17.34	8.01	4.67	0.00
	**Absorbed dose (Gy)**								
15	0.00	0.38 ± 0.16	0.59 ± 0.15	0.84 ± 0.16	1.50 ± 0.16	2.15 ± 0	2.15 ± 0	2.24 ± 0.16	2.34 ± 0.05
20	0.00	0.42 ± 0.18	0.98 ± 0.28	1.63 ± 0.32	2.29 ± 0.16	2.66 ± 0.15	3.60 ± 0.16	3.60 ± 0.16	3.70 ± 0.16
30	0.00	0.94 ± 0.16	1.50 ± 0.15	2.34 ± 0.16	2.99 ± 0.05	4.58 ± 0.16	4.86 ± 0.16	4.95 ± 0.05	5.33 ± 0.33
40	0.00	1.26 ± 0.08	2.79 ± 0.42	4.35 ± 1.13	6.03 ± 0.05	7.15 ± 0.58	7.43 ± 0.62	7.57 ± 0.42	7.71 ± 0.62
60	0.00	1.82 ± 0.31	3.32 ± 0.17	4.72 ± 0.39	5.94 ± 0.32	7.90 ± 0.40	9.39 ± 0.64	10.61 ± 1.29	10.61 ± 0.73
80	0.00	1.85 ± 0.41	3.98 ± 0.16	5.54 ± 0.99	6.62 ± 0.16	10.45 ± 0.35	11.24 ± 0.59	11.71 ± 0.78	12.51 ± 0.15
	**Accumulated dose (%)**								
15	0.00	15.72 ± 6.08	24.22 ± 0.05	36.46 ± 9.85	64.38 ± 10.07	92.31 ± 6.66	92.31 ± 6.66	96.15 ± 6.66	100.00
20	0.00	11.40 ± 7.42	26.82 ± 8.71	44.49 ± 9.86	62.17 ± 6.26	72.25 ± 3.25	79.86 ± 3.58	96.30 ± 5.24	100.00
30	0.00	17.65 ± 3.70	25.43 ± 0.05	44.02 ± 5.06	56.29 ± 3.54	86.06 ± 2.27	91.33 ± 2.60	93.22 ± 5.87	100.00
40	0.00	24.29 ± 1.78	57.83 ± 6.91	65.14 ± 6.16	77.80 ± 7.42	92.69 ± 0.75	96.35 ± 0.37	98.04 ± 2.77	100.00
60	0.00	17.18 ± 2.68	31.29 ± 1.81	54.67 ± 2.98	55.97 ± 3.56	74.45 ± 1.36	88.56 ± 2.98	99.05 ± 0.05	100.00
80	0.00	14.67 ± 3.18	31.37 ± 1.52	44.21 ± 7.19	52.77 ± 5.74	85.53 ± 2.07	89.88 ± 3.48	93.60 ± 5.04	100.00

**Figure 4:**
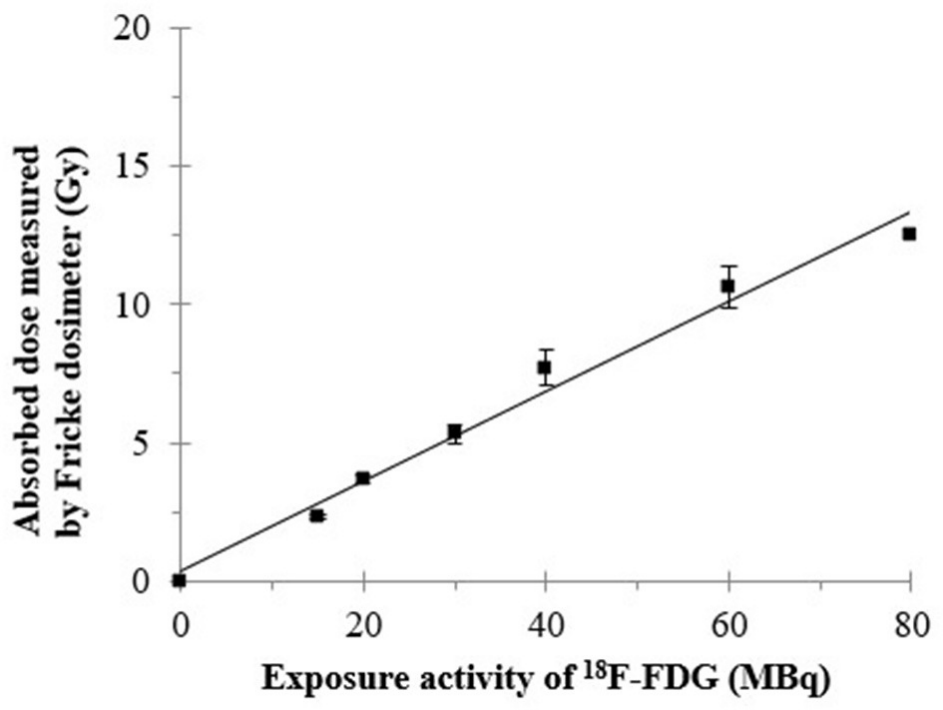
The relationship of radiation dose at the final incubated-time point (1,450 min) measured by the Fricke dosimeter as a function of administered activity of ^18^F-FDG. The dose was measured 1,450 min after addition of ^18^F-FDG into the Fricke dosimeter.

**Figure 5:**
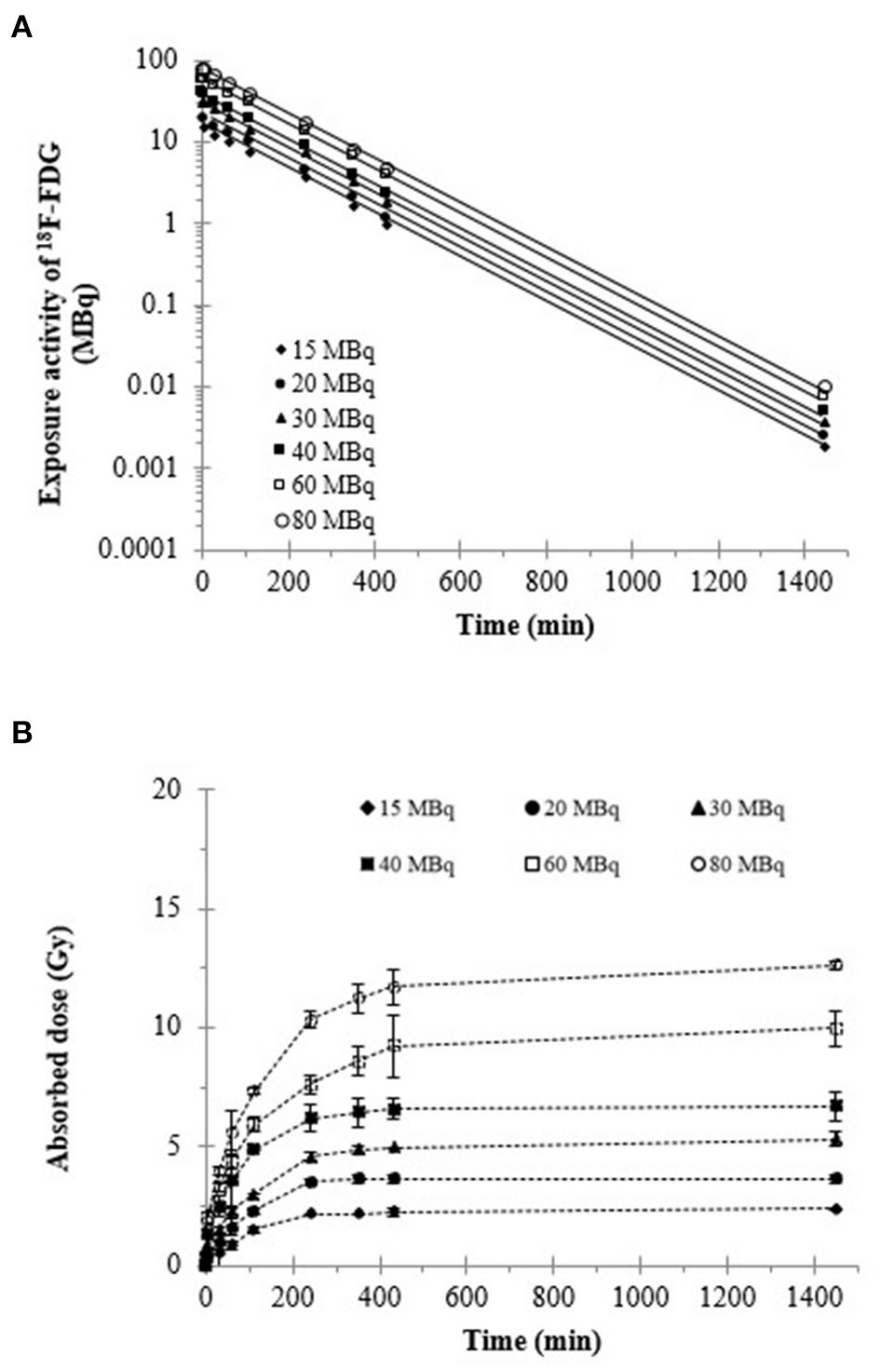
**(A)** Total activity of ^18^F-FDG and **(B)** cumulative dose in the Fricke dosimeter as a function of time. The OD value at 304 nm was measured at the different time points after addition of ^18^F-FDG.

Regarding the physical decay of ^18^F-FDG in the Fricke solution, the doses delivered to the Fricke solutions were calculated from the OD changes in the Fricke solutions containing different activities of ^18^F-FDG; they are shown in Figures 4, 5. The dose delivered to the Fricke dosimeter after 1,450 min of exposure increases linearly as a function of ^18^F-FDG radioactivity (Figure 4). Linear regression showed a R-squared of 0.99. Each result of a given experiment was corrected for the decay of ^18^F-FDG relative to the first OD reading. The linear regression of these data yielded a ratio of dose per exposure activity of about 0.17 ± 0.01 Gy/MBq. Figure 5A shows the exponential decay of the initial activity from time 0–1,450 min after adding 15 to 80 MBq of ^18^F-FDG into the Fricke solution. Figure 5B shows the relationship of accumulated dose in the Fricke dosimeter as a function of time after addition of various activities of ^18^F-FDG.

**Figure 6:**
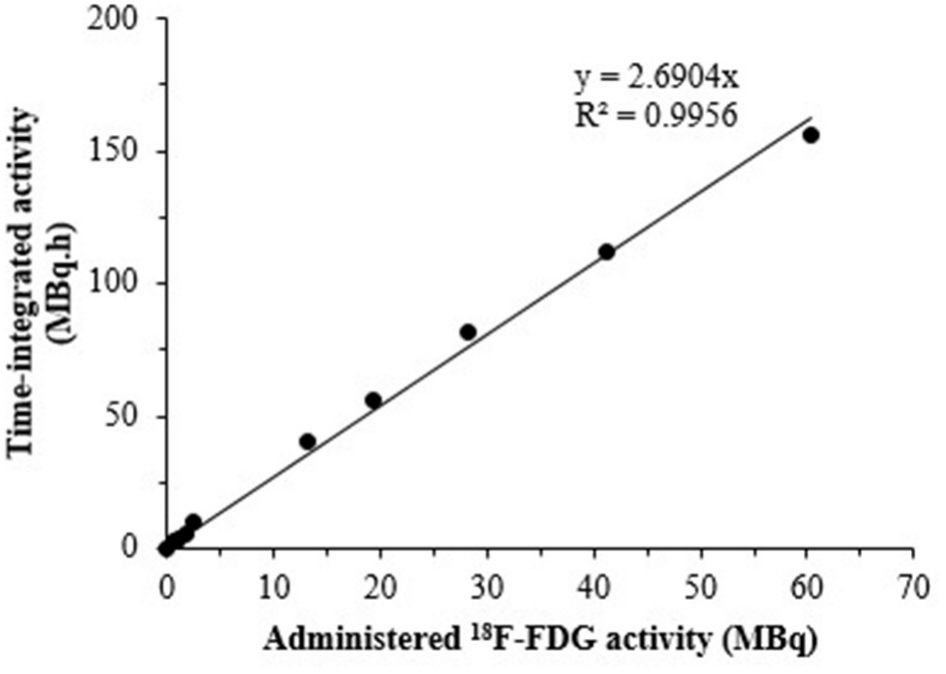
The relationship of time-integrated activity detected by PET imaging and administered activity of ^18^F-FDG.

The relationship of time-integrated activity (MBq.h) with administered activity (MBq) of ^18^F-FDG is shown by the results of Figure 6. As expected, a linear relationship between the time-integrated activity measured by PET and the administered ^18^F-FDG activity was observed. The ratio of total accumulated exposure to administered activity estimated from PET imaging was 2.69 ± 0.06 MBq.h/MBq. The two conversion values of ^18^F-FDG activity to absorbed dose in the Fricke dosimeter (0.17 ± 0.01 Gy/MBq) and of administered ^18^F-FDG activity to time-integrated activity (2.69 ± 0.06 MBq.h/MBq), were later used for calculating a conversion factor of 0.064 ± 0.06 Gy/MBq.h for the dose estimated in the animal dosimetry from PET imaging of the ^18^F-FDG uptake in tissues (Appendix 1).

Table 3 shows the time-integrated activity of ^18^F-FDG that was extracted from the individual tumor tissues and normal organs, allowing a specific estimate of the dose delivered to each tissue/organ from the ^18^F-radionuclide after *i.t*. or *i.v*. administration of the radiotracer. As expected, the radioactivity accumulated at the tumor site is higher after *i.t*. injection compared to *i.v*. injection. Moreover, the relative organ/tumor time-integrated activity values of ^18^F-FDG are lower after the *i.t*. administration compared to the uptake after *i.v*. injection. As the amount of ^18^F-FDG uptake can be correlated with the dose in the tumor and normal organs, the results in Table 4 show that major healthy organs may receive less dose after *i.t*. than after *i.v*. injection. For *i.t*. injection of ^18^F-FDG, the radiation dose calculated in the tumor tissue was higher by more than one order of magnitude than that from *i.v*. injection. Table 4 compares the estimated dose in the tumor tissue after *i.t*. or *i.v*. injection of ^18^F-FDG using the Fricke dosimeter, as a chemical primary standard dosimeter, to those from previous studies using the MIRD method. These data provide support for applying Fricke dosimetry for estimating the dose administered in diagnostic imaging and endoradiotherapy. The absorbed dose in various organs appears to be slightly different when the MIRD method is compared to the proposed Fricke approach. Further investigation would be required to explain this discrepancy.

**Table 3:** Time-integrated activity profiles after *i.t*. administration of 15 MBq ^18^F-FDG (*n* = 3) and *i.v*. injection of 10 MBq ^18^F-FDG (*n* = 1) in a HCT116 nude mouse model.

**Tissue**	***i.t***. ^**18**^**F-FDG**		***i.v***. ^**18**^**F-FDG**	
	**(MBq.h/g)^a^**	**(%IA.h /g)^b^**	**(MBq.h/g)**	**(%IA.h/g)**
Tumor	19.8 ± 0.22	140.36 ± 13.28	0.48 ± 0.02	6.32 ± 2.56
Bladder	11.86 ± 7.92	64.51 ± 32.2	5.36	65.73
Kidney	1.37 ± 0.49	7.72 ± 1.26	2.44	30.11
Liver	0.48 ± 0.3	2.61 ± 1.21	0.35	4.5
Heart	2.6 ± 1.91	8.34 ± 2.51	0.88	10.34
Brain	0.83 ± 0.45	4.59 ± 1.65	0.54	6.35

a *The cumulated activity per gram of tissue (MBq.h/g)*

b *The cumulated percentage of injected activity per gram of tissue (%IA.h/g)*

*For both datasets, values were derived from mean ± SD of biodistribution studies*.

**Table 4:** Mean absorbed dose of ^18^F-FDG in different tissues/organs after *i.t*. injection of 15 MBq ^18^F-FDG (*n* = 3) and *i.v*. administration of 10 MBq ^18^F-FDG (*n* = 1) estimated using the Fricke chemical primary standard dosimeter (Equation 2) and MIRD method (Equation 3).

**Tissue**	**Absorbed dose estimated by Fricke dosimeter (Gy)**		**Absorbed dose estimated by MIRD (Gy)**	
	***i.t*. ^**18**^F-FDG (15 MBq)**	***i.v*. ^**18**^F-FDG (10 MBq)**	***i.t*. ^**18**^F-FDG (15 MBq)**	***i.v*. ^**18**^F-FDG (10 MBq)**
Tumor	0.16 ± 0.05	0.003 ± 0.0001^*^	10.11 ± 1.0	0.45 ± 0.08^*^
Bladder	0.03 ± 0.02	0.013	0.004 ± 0.002	0.04
Kidney	0.02 ± 0.01	0.032	0.01 ± 0.001	0.02
Liver	0.02 ± 0.01	0.005	0.0004 ± 0.0002	0.001
Heart	0.01 ± 0.004	0.007	0.01 ± 0.002	0.006
Brain	0.003 ± 0.002	0.002	0.001 ± 0.0003	0.002

* *HCT116 cells were implanted into both sides of the thigh of the animal (n = 2 for tumor tissue)*.

## Discussion

Nowadays, ^18^F-FDG is the commonly used radiotracer in PET imaging. As a tissue equivalent media, the concentration of ^18^F-FDG in the Fricke solution in this investigation was close to that usually given to a patient undergoing ^18^F-FDG imaging (3 MBq per kg patient weight, up to a maximum of 370 MBq) (31). A typical clinical scan, involving the intravenously administration of 350–750 MBq ^18^F-FDG (32), exposes most tissues in an average patient to a maximum dose of approximately 10 mGy from positron emission (β^+^, *E*_*max*_ = 634 keV) and annihilation photons (γ-rays, 511 keV). The activity of ^18^F-FDG (0–60 MBq) in the present Fricke dosimeter was chosen, first in relation to the clinical observation of the level of accumulated activity of ^18^F-FDG in different tissues. Typically, a patient is injected with 400 MBq FDG (33), and ^18^F-FDG time–activity curves for selected source organs are plotted as activity concentration normalized to administered activity (34). Secondly, this activity was chosen to fit in the range of the maximum detection efficiency of the small animal PET scanner (LabPET8^TM^) at the CIMS, with a linear range from noise equivalent count rate to 60 MBq (35).

The Fricke dosimeter displayed a linear response with exposure to the activity of ^18^F-FDG deposited into the solution. As expected from the mechanism behind the chemical primary standard dosimeter, the dose exhibited a linear relationship with the yields of Fe^3+^ ions in the Fricke solution (22), which is reflected in a change in the OD value, as shown in Figure 2. Therefore, the cumulative exposure to ^18^F-FDG activity in the Fricke solution can consequently be related to the dose of ^18^F-positron irradiation delivered to the Fricke solution. The absorption spectra of non-radioactive FDG irradiated with 80 Gy gamma radiation is expected to have a similar behavior as that of non-radioactive FDG plus 5-15 Gy of gamma irradiation. This is because the response of the Fricke solution is known to be linear over a wide range of doses. In fact, the maximum dose that can be measured accurately is about 400 Gy with low-LET radiation (22). In addition, the Fricke chemical dosimeter can be used in gamma irradiation and electron dosimetry within a dose rate range up to 250 Gy/min (36, 37). We note from the results in Figure 2 that uncertainties in the repeatability of measurements was in the range of 0.51–2.91%, in good agreement with previous reports (38, 39).

Regarding Figure 3B, adding different concentrations of non-radioactive FDG into the Fricke solution results in a very slight reduction of the dose response. This suggests that adding non-radioactive FDG into the Fricke solution may affect the radiochemistry of the Fricke dosimeter. Since the FDG is a glucose analog, Yang et al. suggested that the D-(+)-Glucose has the characteristic of a free radical scavenger, reducing the auto-oxidizing of ferrous ions and stabilizing the dose absorbency response (40). This suggests that adding of non-radioactive FDG could reduce the oxidization of the ferrous ions, leading to the decreased of the yield of the ferric ions as observed in Figure 3B. Further information regarding to the radiolysis mechanism for the degradation of D-glucose in aerated, aqueous solution has been previously reported by Kawakishi et al. (41). Therefore, adding the liquid soluble ^18^F-FDG into the Fricke solution might not change the optical density and extinction coefficient of the Fricke dosimeter for the absorbed dose calculation in Equation (1). However, adding ^18^F-FDG into the Fricke solution, might alter the chemical reaction of the positron radiation with Fe^2+^. The present study takes this issue into account by referring to the negative and positive control group.

Fricke dosimeter response is expressed in terms of its sensitivity, known as the radiation chemical yield or G-value (21). The G-value is defined as the number of moles of ferric ions produced per joule of energy absorbed in the solution. Accurate response of the Fricke dosimeter to ^18^F-FDG is of concern in several features of the technique, including operational dose range, dilution of Fricke dosimeter by the addition of the radioactive tracer, oxygen concentration and careful control of pre- and post-irradiation temperature. Here, the accumulation of dose in the Fricke dosimeter, after addition of various ^18^F-FDG activities, clearly displays an increase with time, until it reaches a plateau as the ^18^F activity is decaying (Figure 5B). The interaction of positron-induced radiolysis radicals with Fe^2+^ yields Fe^3+^ ions and changes the OD of the Fricke solution. Even though the radiation exposure to ^18^F-FDG occurs through a low and continuously decreasing dose rate, radical recombination can occur in the Fricke solution. Such recombination reduces the number of radicals and hence the G-value of Fe^3+^ (40). This effect has been observed with positrons and electrons (42). In addition, O'Leary et al. (43) reported the observation of dose-rate dependence in a Fricke dosimeter irradiated at low-dose rates with monoenergetic X-rays. They observed a dose rate dependence in the G-value for dose rates below 1 kGy/s (40). Moreover, it was suggested that to increase the upper dose range, the yield of ferric ions may be reduced by adding the cupric sulfate to the solution. Such a ferrous cupric sulfate dosimeter would increase the upper range limit to few rads; i.e., in a biologically significant region (44, 45). On the other hand, the lower dose range of the Fricke dosimeter can be extended by increasing the yield of ferric ions. These aspects open opportunities for the future assessment of the internal dose delivered from radiolabelled compounds with the Fricke dosimeter.

The chemical oxidation of Fe^2+^ ions to Fe^3+^ ions in Fricke solution may occur to a small degree without the presence of ionizing radiation due to exposure to oxygen, light or direct contact with any material (46). When the Fricke solution is irradiated, water decomposition occurs and, as a result, hydrogen atoms (H^**.**^) react with oxygen to produce predominantly hydroperoxyl radicals (H^**.**^ + O_2_ → HO_2_^**.**^). With no oxygen present in solution, the number of Fe^2+^ ions oxidized by H^**.**^ is reduced from three to one, significantly reducing the yield. For example, the yields of Fe^3+^ ions after ^60^Co gamma irradiation is about 15.5 ± 0.2 ions per 100 eV in an aerated solution, vs. 8.2 ± 0.3 ions per 100 eV in an anoxic condition (22). For the present study, if oxygen is being depleted sufficiently during the ^18^F-FDG incubation to alter the dosimeter's response. Thus this would be reflected in the departure of the ferric ions yield from the linear trend shown in the Figure 3. A previous study reported no difference of the chemical yield of aerated and oxygen-saturated Fricke solution (47). Oxygenation is not recommended as a routine technique for enhancing the dose response due to the difficulties of identically oxygenating every sample and to the uncertainties in correcting observed absorbance for measured oxygen tension differences (24).

PET imaging provides non-invasive *in vivo* functional imaging, allowing to track molecular mechanisms associated with various diseases. A typical clinical protocol for ^18^F-FDG PET imaging results in a systemic uptake and radiation exposure to most tissues throughout the whole body (19), and thus the local dose depends strongly on radionuclide accumulation in each different tissue. Several dose estimations have been reported based on biodistribution studies in animals or by combining data from animal and human measurements (48, 49). Dose estimation is a very important part of quality assurance programs in medical radiology. However, only few validated and freely available programs are currently available for nuclear medicine (6, 50). The present study combines, for the first time, a chemical dosimeter and PET imaging to evaluate the radiation dose of the ^18^F-FDG PET radiotracer. This technique may provide the opportunity to perform dose estimations, based on the available biodistribution profile data, since the radiation dose delivered to different tissues from PET imaging depends on the PET protocol, the subject's size and organ functions, the amount of administered activity, etc.

With the combination of Fricke dosimeter and PET imaging, the geometric conditions encountered in clinical practice can be taken into consideration and the radiation dose can be related to annihilation radiation detected in PET. In our study, we performed the measurements of the dose in a three-dimensional environment, in both the Fricke dosimetry and PET imaging. We used a subject volume of 3 mL, which provides enough solution to measure the change in the optical absorbance in the Fricke dosimeter. ROIs value (cps/mm^3^) from PET imaging could give the individual cumulated activities with good accuracy. For good PET image quality and reliable quantification, a sufficient number of coincidence events must be detected. Lowering the injected tracer dose means fewer positron-emitting radionuclides and thus less detected 511 keV photon pairs. Therefore, by taking into account this issue of count loss can be compensated by longer PET acquisition times (51). The scan time were calculated according to the previous study of Koopman et al. A= c x w^2^ x T_min_ / t, where c is a constant which is typically 0.0533 (MBq/kg^2^), w is the patient's body weight (in kg), T_min_ is the minimal scan time per bed position needed to be extracted using an image coefficient of variation (COV) of 15 %, and t is the scan time per bed position (s) (52). Our results demonstrate the relationship between dose and the ionizing radiation intensity that takes place during the disintegration process in the Fricke solution. Although the dose in a PET/CT exam is the combination of exposure from the radiotracer and the CT X-ray radiation, in the present study the contribution of annihilation photons (and the few from electron capture) to radiation dose is negligible compared to that of positrons. However, it would be interesting to further extend our approach to evaluate the total radiation dose caused by the radiotracer and the CT scan. Since, only the physical half-life of ^18^F-FDG was considered in the Fricke dosimeter, the dose was estimated by a simple mathematical equation (Equation 1). The value of 0.064 Gy/MBq.h (Appendix 1) was used as a conversion factor to translate the time-integrated activity concentration values of particular target tissues into the dose (Equation 2).

The present study enables to estimate the dose deposition from ^18^F-positron irradiation in the tumor after ^18^F-FDG intratumoral administration. This should allow dose estimation in the tumor and various normal organs. Another advantage of ordinary Fricke dosimetry is the rapidity of dose estimates, necessary for the in-time analysis of biological uptake of radioactive compounds during the PET imaging. The *i.t*. injection of ^18^F-FDG achieves a highly promising target/tissue ratio compared to that of *i.v*. administration of ^18^F-FDG. As shown in Table 4, the estimation of dose in the tumor tissue was 0.16 ± 0.05 Gy after *i.t*. injection of 15 MBq ^18^F-FDG. This is a factor of ~53 higher in dose efficiency compared to *i.v*. administration. However, the distribution of ^18^F-FDG is a highly dynamic metabolic process in terms of biochemical and physical reactions, within the time interval of radioisotope decay. The absorbed radiation dose of ^18^F-FDG in tumor and different organs depends on the uptake kinetics and glucose consumption in tissues. ^18^F-FDG, which is a glucose analog, demonstrates a significant increase in glucose uptake in tumor compared with adjacent tissues (53). This study shows the feasibility of using a Fricke solution doped with ^18^F-FDG for internal dose assessment in clinical nuclear medicine. We hope that it will pave the way for further research on the reconstruction 3D dose mapping by applying the basic principle of Fricke dosimetry.

Here, experimented animals have similar S-values compared to those of animal models reported in other previous studies (Appendix 2), as well as those of the mouse model used in this work and by Taschereau et al. (54). This is not the case for the results of Xie et al. (55), from a normal adult mouse bearing a tumor. Taschereau et al. performed a dose calculation using the GATE Monte Carlo software and a voxel-based mouse phantom containing ^18^F-radiotracers (e.g., ^18^F-FDG, ^18^F-FLT, and Na^18^F) that included a subcutaneous tumor. Our estimate of the dose with the MIRD formalism displays a mean dose in the tumor similar to those previously reported by Taschereau et al. (54). The Fricke dosimeter technique may help to estimate the *i.t*. dose in the tumor tissue and various organs, especially for the short-range of positrons in tissue. However, there are discrepancies of the dose in normal tissues between this study and MIRD, which may be due to the characteristic and large variation in terms of ^18^F-FDG biodistribution in the two mouse models. Some of these discrepancies might be explained by the different pharmacokinetic data, taken for the calculation. In addition, the overall SUV variability is expected to be larger, due to biological factors. For instance, those related to the different types of animal models that may affect the kinetics of the radiotracer. We recorded biodistributions of ^18^F-FDG 120 min after both *i.v*. and *i.t*. injection of 10 MBq/100 μL and 15 MBq/30 μL of the radiotracer, respectively; whereas, Taschereau et al. considered acquiring data of the mouse model from the time of *i.v*. injection of 24 MBq of ^18^F-FDG, up to 90 min. Regarding the time-integrated activity at 90 min, this previous work displays a higher value than that observed in the present study, which may explain the lower dose reported here. It may also be due to the different S-value, which decreases with increasing total body mass, because of larger organ masses. Differences in S-values for organs self-irradiation lie between 2.2 and 3.0%/g difference in body weight (55). The computational model used to assess doses (anatomy, chemical composition, and density) and differences in energy transport may also contribute to the divergences between the doses calculated for normal tissues.

On the other hand, the Fricke approach provides a simple method to rapidly and directly obtain accurate and reproducible internal radiation doses imparted by ^18^F-radiotracers and verify the value of model calculations from fundamental parameters. Moreover, Fricke dosimetry can possibly be extended to other positron-emitting tracers (e.g., ^11^C, ^64^Cu, ^68^Ga, and ^89^Zr) and beta- (e.g., ^177^Lu, ^188^Rh, and ^90^Y) or alpha-emitting radionuclides (e.g., ^211^As, ^213^Bi, and ^223^Ra), as long as the absorbed dose of the emitted radiation can be related to the measured OD values of the Fricke solution. Regarding the radiation track, the stopping power and the range of positrons in biological media, they are similar to those of electrons or beta particles of similar initial energy (56). The Fricke dosimeter response to radiation is a characteristic of its chemical composition that can be guaranteed within a few percent. Previous studies have reported the feasibility of using the Fricke chemical dosimeter for measuring the dose from intimately mixed radionuclides (24). Our study was performed to assess the absorbed doses due to self-irradiation from ^18^F-FDG radioactive solution mixed in the Fricke dosimeter. The Fricke dosimeter exhibited a linear dose response as a function of activity, suggesting its potential applications to assess the absorbed dose in intratumoral targeted therapy. The proposed approach would be suitable for routine measurement of absorbed dose from ^18^F-FDG and any other short-range radiopharmaceuticals to assess the risk from clinical nuclear medicine studies. A calculation technique, called the absorbed fraction method, is available for obtaining Monte-Carlo-based estimates of absorbed dose in certain specific organ system, but some drawbacks still remain (57). Full Monte-Carlo simulations are not recommended for routine clinical use due to complex calculations and relative long computational times (roughly 3 h for about 10 million simulations) (58). For voxel-based dosimetry in analogy with MIRD, variances are often within a few percent and are not considered relevant in a clinical setting (59).

Since exposure of the Fricke dosimeter to ^18^F-radiation results in about 97% of positrons emitted by ^18^F-FDG and subsequent annihilation photons (γ-rays, 511 keV), a local energy deposition method for dosimetry calculation was considered in the present study. Therefore, the method will yield a very accurate estimate of the self-irradiation dose due to positron but neglects annihilation photons. The much shorter-range positrons take a random path though matter losing its kinetic energy via ionizing events, inelastic scattering or in producing excited molecular species and free radicals, which thermalize within 10^−11^ seconds (60). Diffusion after thermalization until annihilation involves distances of only about hundreds of nanometers. Positron lifetimes in molecular media are nearly about 400–500 picoseconds after which they may annihilate as free positrons (61).

Although the theory of local energy deposition holds true for certain particles (alpha, beta, positron or Auger electrons), it does not apply for gamma emissions or secondary photons due to the longer penetration depth and the much lower density of deposited energy. However, if one's primarily interest is assessing the local self-dose tissues or organs, then this method is fairly accurate for a quick analysis like in toxicity studies (62). The gamma irradiation cross-fire effect between tumor and organs or between organs is considered marginal in targeted radionuclide therapy (63). Concerning the biological response after intratumoral administration to a low-dose of 634 keV β+ and 511 keV γ-rays, previous studies reported radiation-induced DNA damage and the relative biological effectiveness of ^18^F-FDG in animal model (19). The relative biological effectiveness of radiation quality from ^18^F-FDG, with respect to malignant tissues is ~1. Taylor et al. also investigated the biological response of non-cancer endpoints. They found that the 10 mGy PET treated animals had significant reduction in kidney lesion, indicating that a higher absorbed dose (20 ± 0.13 mGy), relative to the whole-body average, which occurs in specific tissues may not be detrimental. In other words, the mixed radiation quality and/or low-dose rate from PET scans is less damaging than equivalent doses of gamma radiation (19).

A typical clinical scan involving the administration of 350–750 MBq ^18^F-FDG (33), exposes most tissues in the average patient to a maximum absorbed dose of approximately 10 mGy from positron emission and annihilation photons. The critical organ after ^18^F-FDG administration is the urinary bladder, which is exposed to 0.16 mGy/MBq in adults; although this can be reduced with patient hydration and increased patient voiding frequency (64). In addition, tissues with increased uptake of the radiopharmaceutical receive higher absorbed doses than the whole body average including the brain (10–36 mGy), heart (16–51 mGy), kidneys (7–23 mGy) and bladder (13–233 mGy) (20). The accurate determination of the radiation dose to the bladder wall from ^18^F-FDG is important, because the bladder is the critical organ in radionuclide targeted studies. The radiation dose to the bladder wall from injected ^18^F-FDG can be estimated using both a dynamic bladder model and the conventional MIRD model. Previous study by Dowd et al., has evaluated the radiation dose to the bladder wall from 2-[^18^F]fluoro-2-deoxy-D-glucose in adult humans (65). The factors that play the largest role in the calculation of the dose to the bladder wall using the dynamic method were urine production rate, initial bladder volume, and the residual volume in the bladder after voiding. They also mentioned that an exact determination of a bladder time-activity curve was not extremely important to the calculation of the dose to the bladder wall from the bladder contents. They showed the relationship between the dynamically estimated bladder dose as a function of bladder size at injection. When using estimates based on the dynamic model at low initial bladder volumes, the dose to the bladder was considerably increased. For the MIRD method yields an average result of 0.39 and 0.35 rad/mCi for those subjects whose bladder volumes at injection, were calculated to be 75 and 550 ml, respectively.

The dose in different tissues deduced from the dose delivered from ^18^F-FDG to the Fricke solution can be obtained by calculating the correction factor, which refers to the number of annihilation events, and thus positrons, detected in PET imaging. The dose measured by the Fricke dosimeter and PET imaging was compared to that calculated with the MIRD formalism. Fricke dosimetry displays slightly higher values of the doses delivered to normal tissues than those calculated by MIRD. A large difference (approximately two orders of magnitude for *i.v*. and *i.t*. ^18^F-FDG administration) of such doses delivered to tumor tissues is reported in Table 4. These disparities are significant and must be discussed. One possibility is that with the Fricke dosimeter, the dose was estimated by a simple mathematical equation regarding radioactive decay, integration with time, but with no changes in biological distribution. Notably, relying directly on Fricke dosimetry, while not considering the time course of ^18^F-FDG in tissues, may have a pronounced influence on dose estimates. Moreover, considering only ^18^F-FDG, the accumulated dose to the Fricke solution is mainly due to activity and total deposited energy of positrons in the Fricke dosimeter. However, only about 10% of the number of photons produced during the annihilation process can be efficiently detected by clinical PET scanners (66). In addition, the coincidence detection efficiency (or absolute sensitivity) of the LabPET8 scanner used in this study is only about 2.6% (28). Inaccurate calibration of the absolute sensitivity of the PET scanner could make a significant difference for the estimated final absorbed dose between these two techniques. Furthermore, Fricke dosimetry assumes that the activity of ^18^F-FDG is distributed uniformly within each organ, as will therefore be the emitted energy. This could contribute to a much higher local ^18^F-FDG concentration than with the organ average MIRD calculation.

In addition, there are various factors influencing the response of Fricke dosimeter to ionizing radiation, including oxygen, ferrous ion concentration, sulfuric acid concentration, purity of reagent and cleanliness of glassware, dose rate, LET and temperature (22). Previous studies have evaluated the dose response of chemical FBX dosimeter to ^99m^Tc radionuclide (gamma-ray emission energy is 142 keV) in different geometries (e.g., cylindrical, rectangular or spherical) of glass containers with nominal volumes of 10, 100, and 1,000 mL (24). They observed that there were no differences between the container geometries. The dose response curves were identical for all tested geometries and were linear up to a high activity of 74 GBq. In addition, they also reported results comparing FBX response data for rectangular container and nominal dosimeter volumes of 10, 100, and 10,00 mL, where the slopes of linear response curves were 0.02422, 0.00366, and 0.00048 absorbance/mCi, respectively. The dosimeter volumes were different by a factor of 10, but the dose responses as measured by the slopes, by comparison of the mean absorbed dose per unit cumulated activity (S-value) were different by factors of about 7–9 between the larger volumes and about 5–7 between the smaller volumes. The mean free path in water of 140-keV photons of energy from ^99^Tc and 511 keV photons from ^18^F is about 7 and 10 cm, respectively (67). From this consideration, further investigations could experimentally validate the influence of volume on the dose response in the Fricke dosimeter from ^18^F-FDG radiopharmaceutical homogeneously dispersed throughout the volume of any arbitrary container. Moreover, Fricke dosimeter could be designed to evaluate the dose from irregular and complex irradiation tumor geometries to distant tissues. Monte-Carlo simulations would be useful validate our results, for instance, by considering the addition of ^18^F-FDG in several volumes of Fricke solution.

Finally, we would like to mention that neither the MIRD formalism (30) nor direct dose measurement with the Fricke dosimeter provide the details of the energy distribution of radiation energy from the positrons and those of the ensuing damage to biomolecules and targeted cells. Since the uptake of a radiotracer in a target tissue is particularly non-uniform, averaging the dose over the entire tissue by the Fricke approach may be an oversimplification of the actual energy deposition pattern, as well as the crossfire effect (68). More details could be provided by elaborate Monte Carlo code calculations that would incorporate the necessary parameters (2). These include principally the interaction cross sections of secondary electrons and reaction rate constants of all chemical species generated in the biological medium by the positrons. Secondary electrons have a wide energy distribution, peaking around 10 eV (69). At such low energies, however, their cross sections to damage biomolecules are dependent on the state of aggregation of condensed matter (70) and hence their availability remains limited (71). In any case, additional information from elaborate Monte Carlo simulations should provide a complete and more accurate view of the influence of charge particle contributions to the absorbed dose in Fricke dosimetry and hence establish a better correlation with the activity assessed by PET imaging.

## Conclusion

This study explains the feasibility of using a Fricke solution as a primary standard chemical dosimeter for measuring the dose delivered by positron-emitting radiotracers (e.g., ^18^F-FDG). The technique has the potential for estimating the dose distribution *in vivo*. Considering the remarkable simplicity of the Fricke dosimeter, it should be possible to apply other strategies (e.g., gel-based chemical dosimetry) to develop suitable systems capable of multi-dimensional dose mapping. This can be helpful in the development of internal dosimeters to avoid under- or over-exposure of patients, not only by ^18^F-radiotracers, but also by other radionuclides used in PET. Moreover, since electrons and positrons have essentially the same scattering properties, the method should also be applicable to short-range electron emitters (e.g., Auger electron sources). This may have important implications in targeted cancer treatments, particularly to more accurately determine radiotherapeutic doses delivered to cancer cells and collateral doses to healthy tissue.

## Data Availability Statement

The raw data supporting the conclusions of this article will be made available by the authors, without undue reservation.

## Ethics Statement

The animal study was reviewed and approved by the Université de Sherbrooke Animal Care and Use Committee (protocol number: 235-14B).

## Author Contributions

TT: designed, conducted and analyzed the experiments, and wrote and revised the manuscript. EB-S: helped with ^18^F-experiments. BG, RL, BP, and LS: reviewed and revised the text and figures. All authors contributed to the article and approved the submitted version.

## Funding

This work was supported by the Canadian Institutes of Health Research (Grant # MOP-81356) and the Centre de Recherche du CHUS (CRCHUS). BG, RL, BP, and LS are members of the CRCHUS supported by the Fonds de la Recherche du Québec - Santé.

## Conflict of Interest

RL is a founder and Chief Scientific Officer of IR&T Inc. The remaining authors declare that the research was conducted in the absence of any commercial or financial relationships that could be construed as a potential conflict of interest.

## Publisher's Note

All claims expressed in this article are solely those of the authors and do not necessarily represent those of their affiliated organizations, or those of the publisher, the editors and the reviewers. Any product that may be evaluated in this article, or claim that may be made by its manufacturer, is not guaranteed or endorsed by the publisher.
