# Supplementary material for: Estimation of the Internal Dose Imparted by 18F-Fluorodeoxyglucose to Tissues by Using Fricke Dosimetry in a Phantom and Positron Emission Tomography
Source: Front Nucl Med. 2022 Feb 14;2:815141. doi: 10.3389/fnume.2022.815141 (PMC11440868; doi:10.3389/fnume.2022.815141)
Supplement: Supplementary file 1 [file Data_Sheet_1.docx]

**Appendix 1: Conversion factor for dose calculation of ^18^F-radiation using the Fricke chemical primary standard dosimeter.**

For instantaneous uptake with no biologic excretion, the total cumulated activity (Ă) can be calculated as indicated in equation [A1], presenting a relationship of cumulated activity *Ă* (MBq.h) per administered activity *A*_0_ (MBq) (Figure 6).

*Ă* = 1.44 * *A*_0_ * *T*_p_ [A1]

where

*A*_0_ is the initial activity (in MBq) and *T*_p_ the physical decay half-life of the source (in hours).

Therefore, by considering Figure 4 and 6, we can have a conversion factor derived as follows:

Fricke dosimeter: *f*(Gy) = *a*_F_*X* + *b*_F_ = 0.1687*X*

PET imaging: *f*(MBq.h) = *a*_P_*X* + *b*_P_ = 2.6904*X*

Conversion factor: *C* = *a*_F_ /*a*_P_ = 0.1687/2.6904 = 0.064 (Gy/MBq.h)

By using this conversion factor of 0.064 Gy/MBq.h, total absorbed dose of ^18^F-radiation can be calculated using equations [2] as indicated in the main text.

**Appendix 2:** S-values from ^18^F-radionuclide and organ mass in a 25-30 g mouse model.

S-Value is the dose factor (mean dose per time-integrated activity in the source region).^45^ The table A1 below displays the mass of different organs as well as a comparison of the S-value used for the MIRD calculation in this study and those reported in previous studies by Xie et al (55), Dieudonné et al (72), and Keenan et al (73).

**References**

1. Dieudonné A., Hobbs R.F., Bolch W.E., Sgouros G., Gardin I. (2010). Fine-resolution voxel S values for constructing absorbed dose distributions at variable voxel size. J Nucl Med.  51:1600-1607.
2. Keenan M.A., Stabin M.G., Segars W.P., Fernald M.J. (2010). RADAR realistic animal model series for dose assessment. J Nucl Med. 51:471-476.

**Table A1** The mass and S-value of different organs used for the MIRD calculation

| Tissue | Mass (g) | S-value (mGy/MBq.h) | | |
| --- | --- | --- | --- | --- |
|  |  | Keenan *et al.* | Xie *et al.* | This work |
| Tumor | 0.1 |  |  | 20 |
| Bladder | 0.039 | 0.459 | 0.456 | 0.456 |
| Kidney | 0.206 | 0.097 | 0.0945 | 0.095 |
| Liver | 0.247 | 0.018 | 0.018 | 0.018 |
| Heart | 0.133 |  |  | 0.127 |
| Brain | 0.065 | 0.067 | 0.065 | 0.065 |
